# Which factors increase informal care hours and societal costs among caregivers of people with dementia? A systematic review of Resource Utilization in Dementia (RUD)

**DOI:** 10.1186/s13561-021-00333-z

**Published:** 2021-09-18

**Authors:** Renira C. Angeles, Line I. Berge, Marie H. Gedde, Egil Kjerstad, Maarja Vislapuu, Nathalie G. Puaschitz, Bettina S. Husebo

**Affiliations:** 1 NORCE Norwegian Research Centre AS, Department of Social Science, Health Services and Health Economics Research Group, Bergen, Norway; 2 Centre for Elderly and Nursing Home Medicine, Department of Global Public Health and Primary Care, University of Bergen, Bergen, Norway; 3 NKS Olaviken Gerontopsychiatric Hospital, Askoy, Norway; 4 Haraldsplass Deaconess Hospital, Bergen, Norway; 5 Centre for Care Research West, Western Norway University of Applied Sciences, Bergen, Norway; 6 Municipality of Bergen, Bergen, Norway

**Keywords:** Resource utility dementia, Cost of illness, Cost-effectiveness, Informal caregivers, Family caregivers

## Abstract

**Background:**

Nearly 19 million people across OECD countries are living with dementia, and millions of family caregivers are affected by the disease. The costs of informal care are estimated to represent 40–75% of the total dementia cost exceeding formal care time and medical costs.

**Objective:**

To conduct a systematic review to evaluate the methodological quality and factors associated with high informal care hours per month that increase societal costs, and to identify what type of interventions may alleviate the entire burden of informal and formal caregiving.

**Methods:**

The systematic review was registered at PROSPERO (15.12.2020). A search in Medline, Embase, PsycINFO, and web of science for observational studies, cost-effectiveness, and cost of illness (COI) analyses on resource utilization in dementia (RUD) was conducted on 1 December 2020. Our inclusion criteria included a requirement that studies had to use the original RUD, RUD-FOCA or RUD lite in terms of hours or days per month, and costs as primary or secondary outcome, OECD countries, within the last 20 years and a sample population comprising persons with dementia (PwD) ≥65 years and their caregivers. We followed the PRISMA, GRADE, PICO guidelines and Drummond criteria to assess the methodology and quality of the studies.

**Results:**

Of 307 studies**,** 26 cross-sectional and 3 longitudinal cohort studies were included in the analyses. Two studies had a randomized controlled trial (RCT) design. The methods and cost categories in each study varied widely. Disease severity, caregiver factors, and behavioural and psychological symptoms of dementia (BPSD) were associated with high informal care hours and societal cost. One RCT found no effect of a non-pharmacological intervention on informal care hours, yet another RCT found a cost-effective impact of an in-home respite care programme reducing informal care burden and costs.

**Conclusion:**

The divergent use of the RUD components within included studies encourage more harmonized analyses. There are only two RCTs on RUD, one of which shows a significant treatment effect. Larger sample sizes and longer follow-up periods are required in future RCTs with dedicated focus on cost-enhancing and resource intensive factors such as disease severity and BPSD. Novel interventions must diversify between caregiver and PwD groups.

**PROSPERO registration:**

CRD42021226388.

## Introduction

Nearly 19 million people across OECD countries are living with dementia, and millions of family members who provide care and support throughout their lives are directly or indirectly affected by the disease. Total costs, i.e., costs related to informal care (unpaid care provided by family and others), direct costs of social care (provided by community care professionals and in residential home settings) and direct costs of medical care (treatment costs and other conditions in primary and secondary care), in dementia were estimated to be US$279.6 bn in 2000, US$ 604 bn in 2010 [1, 2], $948 bn in 2016, with an annual growth rate of 15.9% [3]. Since 2010, the total costs have grown by 35% [4]. Globally, it was estimated that costs related to dementia passed USD 1 trillion in 2018, and the number was expected to triple the next 20 years [5]. As people not only live longer, but also spend a longer time span of their last years at home, the caregiver burden of dementia rises, as well as the need for family members to provide care 24 h a day [5].

Informal care is the care provided by family caregivers, and formal care by professional staff. The more physical disabilities and cognitive decline, the more formal and informal care complement each other [6–8]. Informal care costs constitute 40–75% of total dementia cost, exceeding formal care costs, and total medical costs [7]. Formal care is only the second largest cost component of the total cost [9, 10].

A large number of informal care hours are associated with lost productivity or leisure time by informal care givers [11]. Worsening physical and mental health [12], poorer quality of life for caregivers [12], institutionalization [12], increased financial burden [13], and disease progression [14] are other factors associated with informal care. Thus, increasing caregiver hours affect informal care cost [1, 15] in several different ways. The economic aspects of dementia are a major public health policy concern [16], particularly family relations that establish the infrastructure and supply of informal care of persons with dementia (PwD).

Resource Utilization in Dementia (RUD) is an assessment tool developed and validated to evaluate resource use for PwD. RUD has been proven comparative across countries with different health care systems [10, 17] and provides the number of hours and days per month of care within activities of daily living (ADL), instrumental activity of daily living (IADL) and supervision by informal caregiver. RUD is used in trials, observational studies, and economic evaluations, and is thus a useful source for health policy makers, decision making and planning of dementia care services by making distributions of several cost components transparent [18].

Patient factors such as behavioural and psychological symptoms of dementia (BPSD), disease severity and progression, and caregiver attributes such as age, socio-economic status, employment- and situation, and the ability of the caregiver to deal with the burden of caring are key factors influencing RUD. Economic evaluations using different valuation methods are one of the major concerns when estimating the cost of dementia [19].

In this systematic review of the literature, we investigate the resource utilization in dementia care with and without explicit economic evaluations, as well as publications with solely economic evaluations using RUD as cost input and primary outcome. Although, there are some studies reviewing the economic evaluation of dementia [10, 19, 20], this is the first to review both economic evaluations and research papers jointly. The benefits of focusing on RUD studies is the gain in comparability and in-depth methodological analyses. In previous economic evaluation studies, cost estimates vary depending on the method of valuation for informal care [21]. Following the PICO framework [22], the review aims to investigate the following questions.

1. What factors are more strongly associated with high caregiver burden and increased societal costs?
2. Are there specific health service interventions that are more beneficial in terms of reducing caregiver burden and societal cost levels?
3. What is the methodological quality of the economic evaluations of RUD studies?

## Methods and analysis

### Protocol and registration

This study is conducted in accordance with the PICO and PRISMA guidelines for reporting of systematic reviews and meta-analyses, [23] and is registered at PROSPERO (CRD42021226388). The protocol can be accessed at https://www.crd.york.ac.uk/prospero/#recordDetails.

#### Search strategy

##### Electronic databases

We did a systematic search in Medline, Embase, APA PsycINFO, and Web of Science for the period 2000–2020 on 1 December 2020. The following combined search terms were used: RUD OR “resource utility dementia” OR “resource utility in dementia” OR “RUD-FOCA” OR “RUD-formal care” OR “resource utility dementia-formal care” AND caregiver OR “care giver” OR carer OR “care burden” OR “formal care” OR “informal care” OR “nursing home*” OR “care institution”, “health policy” OR “health care policy” OR HTA OR “health technology assessment” OR “health economics” OR “cost-benefit” OR “cost benefit*” OR “cost analysis” OR “cost assessment”. The search terms were adjusted to the specific databases and consisted of free-text search.

##### Language

We included studies published in English.

##### Study selection

The reference search and part of the selection method was done by the university librarian RKL and first author RCA. Search results were independently reviewed and screened by MHG and RCA at three levels: titles, abstracts, and full text papers. All studies including cost-estimation and observational analyses not using RUD, RUD-FOCA, RUD Lite were eliminated. RUD Lite is a shorter version of the RUD, which focuses mainly on the patient’s resource use more than the caregiver’s [24]. The RUD and RUD-FOCA instruments measure the informal care time for family caregivers in home-dwelling, and nursing home PwD [25]. The selection criteria of cost-effectiveness and cost of illness (COI) studies were practised according to Drummond and Jefferson [26]. All published protocols were eliminated by RCA and MHG.

##### Study design and participants

We included research papers on informal caregiving to PwD living either at home or in nursing homes. The inclusion and exclusion criteria are displayed in Table 1. We included papers with RCT design, observational analyses, and economic evaluations such as cost-effectiveness and COI studies. Studies that include both observational analyses and economic evaluations assess the relationship between demographic and clinical factors, and total costs of dementia. Reviews, editorials, and conference papers were excluded. Further, we included studies that recruited participants ≥65 years. Studies that do not report age criteria but nevertheless reported a mean sample age higher than 75 were included. We included studies within the OECD countries, including cross-country studies with non-OECD countries in the sample, studies from 2000, and studies that had RUD as primary or secondary outcome. As nursing home dementia patients have significantly lower informal care hours [27] research papers studying formal care with the RUD-FOCA instrument *that did not assess informal care* were excluded.

**Table 1:** Review inclusion criteria

	Inclusion	Exclusion
**Setting**	Community-dwelling, nursing home	
**Age**	Participants ≥65 years	
**Study outcome**	RUD in terms of hours per month, days per month, and cost as primary or secondary outcome	Informal care hours not based on RUD. Research papers with a nursing home setting which did not assess informal care
**RUD versions**	Original RUD, RUD-FOCA, RUD Lite	
**Type of analyses**	RCT design, observational analyses, economic evaluations	Descriptive
**Type of paper**	Research papers with the following design: longitudinal, cross-sectional, cross-country, economic evaluations	Review, editorial, conference papers
**Geography**	OECD countries	
**Year**	2000–2020	

##### Outcomes

The focus of this review was to identify factors affecting both formal and informal care hours per month measured by the original RUD and RUD-FOCA, as well as costs at different levels (individual, society). The RUD and RUD-FOCA instruments measure the informal care time in terms of hours and days per month for family caregivers in home-dwelling and nursing home PwD [25]. Both assessment tools comprise three parts: instrumental activities of daily living (IADL) covering assistance with medication, financials, transportation, grocery shopping and cooking meals, personal activities of daily living (PADL or ADL) such as assistance with personal hygiene, and the extent of mobility [17, 18, 25, 28, 29]. The RUD instrument assesses informal care on monthly hours of advising and supervision, i.e., surveilling dangerous events.

##### Quality assessment

To evaluate the quality of the observational studies and/or the economic evaluation, we followed the recommendation checklists of international health economic guidelines [26, 30], and for COI studies we followed the COI evaluation checklist [31, 32]. Further, we followed the quality assessment recommendation according to the GRADE [33]. GRADE provides criteria for rating the quality of evidence that includes study design, risk of bias, imprecision, inconsistency, indirectness, and magnitude of effect. Following Drummond et al. [34] and Molinier et al. [32], we use the following ten points to assess the studies including economic evaluations: clear definition of illness, comprehensive description of competing alternatives, disaggregation of costs, identification of all important and relevant costs and consequences for each alternative, costs and consequences measured accurately in appropriate physical unit, unit costs appropriately valued, careful explanation of the methods adopted, discounting of costs, performance of sensitivity analysis, inclusion of all issue of concerns in the presentation and discussion.

## Results

### Number and type of studies

**Fig. 1:**
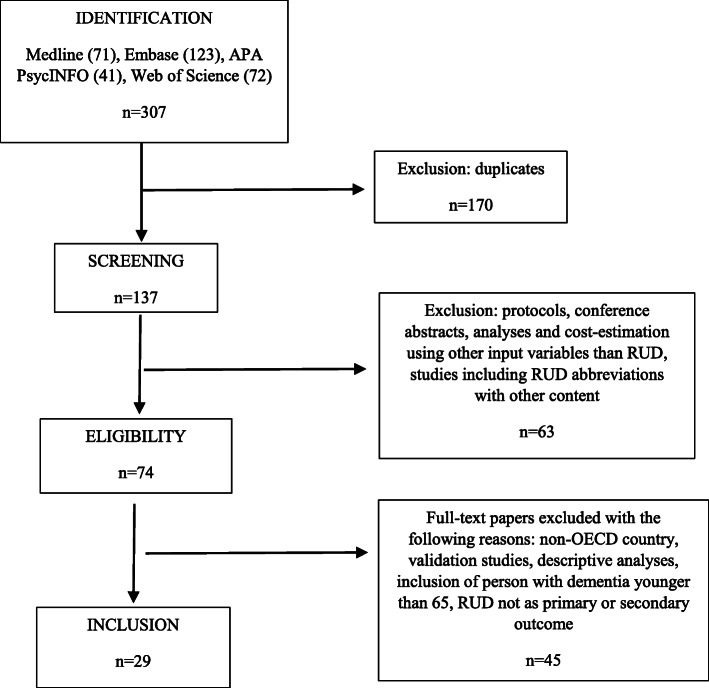
Flow diagram of RUD study selection

The database searches retrieved 307 records. (Fig. 1). We excluded 170 duplicates and eliminated 63 studies in which RUD in the title did not refer to Resource Utilization in Dementia. In addition, from the remaining 74 articles, we excluded protocols and conference abstracts, descriptive studies, including economic evaluations using other input than RUD, and ended up with 29 papers within the period 2000–2020 that were included in the systematic review (Fig. 1). Three studies were cohort studies, 26 were cross-sectional and two had an RCT design. Nineteen studies combine observational analyses and economic evaluation based on RUD, two studies had only cost-estimation, and eight studies include only observational analyses.

### Non-cost evaluation studies

Table 2 summarizes the eight observational and RCT studies informing the type of study, setting, age, dementia stage, research methods and main conclusions. Seven studies [35–40] defined dementia disease with the Mini Mental State Examination Scale (MMSE) (range: 0–30) [41]. One study, by Sköldunger et al. [42], used the Global Deterioration Scale for assessment of primary degenerative dementia (GDS) (range: 1–7) [43]. Among the studies in a home-dwelling setting [35–38, 40, 44] there was no specified dementia disease in all 7 studies. There was one RCT study by Luttenberger et al. [39] (*n* = 119) that followed caregivers for persons accommodated in nursing homes within a six-month period. This study found no impact of a non-pharmacological multicomponent therapy including 24 h assistance to ADL and IADL care, on informal caregiver hours within the intervention period. Despite the null finding, there were some limitations that increased the risk of bias. The lack of blinding may have increased distortion. Despite the strength of a control group, the follow-up period was six-months. Hajek et al. [45] is one of the three longitudinal studies assessing caregiver time for 126 community-dwelling PwD at 6-month intervals over a 1.5-year period, in total 4 assessments. The magnitude of the effect of dementia severity on total caregiving time (formal and informal care) increased by 39.4% per month at 1% level of significance; 68% of the effect was informal caregiving time, which was deemed to have high clinical relevance. Married patients received more total caregiving time (+ 33%), and 72.5% of the effect was informal caregiving hours. Neubauer et al. [36] found a significant effect of the PwD’s functional level for both primary caregiver and secondary caregivers, but the difference in the effect between the groups was rather small (0.07 and 0.10, *p* < 0.05). The t-test showed that informal care time by a secondary informal caregiver was underestimated by 14%. A strength of the study was the comparison with formal care. Parrota et al. [40] found a 1.7 increase in supervision hours per day when PwD had depressive symptoms. Depression was defined with the Cornell Scale for Depression in Dementia (CSDD) [46]. The strength of the study is the magnitude of the findings, also including the explicit discussion of confounders, and the comparison with other caregiver burden measurements such as the Zarit caregiver burden scale [47], while a limitation was that it did not include other dimensions of RUD. Two studies investigated disease progress by MMSE using nursing home population comparing people with and without dementia. One of them, by Nordberg et al. [38] found no effect on the level of cognitive functioning on informal care time, while the other, by Nordberg et al. [37] found a significant difference between formal care and informal care users. A major limitation was that these bivariate analyses are not adjusted for other factors associated with both MMSE and care times, such as ADL function and neuropsychiatric symptoms. Sköldunger et al. [42] found that both dependency and disease severity increased informal care hours by 1.5 h per week which was of low magnitude. Finally, a cohort study by Teipel et al. [35] found a significant association between total score of the Neuropsychiatric Inventory (NPI) [48] and increased caregiver burden. This finding was robust to separate items in the NPI instrument, and by clustering within different GPs. A strength of the study was the use of all three parts of RUD assessing hours per month; supervision, IADL and ADL, and the analyses incorporated a comparison of the effect of different psychotropic medication to different NPI-symptoms (items) as an alternative analysis. Among the eight non-cost evaluation studies, one study used RUD-FOCA [39], five studies used all measurements of RUD [36–38, 42], one study, by Teipel et al. [35] excluded supervision, and one study only, by Parrotta et al. [40], used supervision.

**Table 2:** RUD studies with observational non-cost analyses (*n* = 8)

Author, year, country, source number	Type of study	Setting, age PwD (age CG), dementia stage	Method / data analysis	n	Dementia severity definition	Conclusion
Teipel et al. (2015) Germany [35]	Cohort study	Community-dwelling; 79.1; MMSE 21–23	t-tests	176 dyads	MMSE	Neuropsychiatric symptoms in a primary care cohort with dementia were associated with resource utilization and distress of caregivers.
Sköldunger et al. (2018) Sweden [42]	Cross-sectional	Nursing home population; 86; GCS severe 15.7%, moderate 25.7%	OLS^a^, GLM^b^	4831	Gottfries cognitive scale (GCS)	Impaired cognitive function and functional dependency increases the resource use in nursing homes.
Neubauer et al. (2008) Germany [36]	Cross-sectional	Community-dwelling; 80 (59.4); MMSE 18.6, NOSGER^c^ 19.9	OLS, logit	313	MMSE, NOSGER	Previous studies underestimated costs of informal care because the time of informal caregivers other than the primary caregiver was not considered.
Hajek et al. (2016) Germany [45]	Longitudinal	Community-dwelling; 85 at baseline, MMSE 20.1	OLS RE^d^	126	CDR^e^, GDS, MMSE	Informal caregiving time strongly increased with dementia severity.
Nordberg et al. (2005) Sweden [37]	Cross-sectional	Home-dwelling; 81.8; MMSE 25	Bivariate regression	740	MMSE, CDR	There is a stronger relationship between the severity of the cognitive decline and the amount of informal care rather than formal care.
Nordberg et al. (2007) Sweden [38]	Cross-sectional	Community-dwelling; 84.6; MMSE 13	Tobit regression	176	MMSE, CDR	There is a variation in time use in institutional settings due to differences in ADL dependency, but also whether dementia is present or not. This variation has implications for costs of institutional care.
Luttenberger et al. (2012) Germany [39]	Cross-sectional, longitudinal	Nursing home; 84.7; MMSE 15.2	Regression	160	MMSE	The 6-month non-pharmacological intervention improved dementia symptoms in nursing home residents, especially in social behavior and IADL capabilities, but no effect was seen on informal care time.
Parrotta et al. (2020) Cross-country^f^ [40]	Cross-sectional	Community-dwelling; 81.9 (62); MMSE 12.5	Regression	1223	MMSE	Depression in PwD is associated with an increased burden and distress of informal caregivers and a reduction of their quality of life.

*PwD* people with dementia, *CG* caregivers. ^a^ Ordinary Least Square regression, ^b^ Generalized Linear Model, ^c^ Nurses’ Observation Scale for Geriatric Patients [84], ^d^ Random Effect, ^e^ Clinical Dementia Rating [69] ^f^ Finland, Estonia, Germany France, the Netherlands

### Combination studies – economic evaluation, observational- and RCT studies

Table 3 summarizes 21 studies with economic evaluations (cost-estimations) and provides information on the type of study, sample size, research methods, setting, mean age of the PwD and their caregivers, and conclusions. Vossius et al. [49] was the only study providing longitudinal data with a 2year period and a 6 month interval. Except for Boström et al. [50], Nakabe et al. [51, 52], and Hojman et al. [53], all studies in Table 3 used MMSE or CDR to define the stage of dementia in the sample. Boström et al. [50] had preselected participants with either dementia with Lewy Bodies (DLB) or Alzheimer disease. Vandepitte et al. [54] and Carter et al. [55] used pure cost-estimations whereas Carter et al. used COI analyses and Vandepitte et al. cost-effectiveness of a Belgian randomized controlled trial of home-dwelling PwD. Following Drummond et al. [34] and Molinier et al. [32], Table 4 lists all cost studies providing information on the type of health care system, valuation year, currency, cost variables identified, perspective, the unit for which the costs are provided, total societal costs, and the share of informal costs to total costs.

**Table 3:** RUD studies with observational analyses and economic evaluation (*n* = 21)

Author, year, country, source number	Type of study	Setting, age PWD (age CG), dementia stage	Method / data analysis	n	Dementia severity definition	Conclusion
Michalowsky et al. (2018) Germany [72]	Cross-sectional, economic evaluation	Community-dwelling; 80.2; MMSE 22.8	OLS, cost-estimation	425 PWD, 254 dyads	MMSE	Costs of care doubled over the stages of dementia. For all cost categories, deficits in daily living activities were major cost drivers.
Darba et al. (2015) Spain [68]	Cross-sectional, economic evaluation	Community-dwelling; 76.2 (59,6); median CDR 1	GLM, cost-estimation	343	CDR	The costs of care for people with AD in Spain were substantial, with informal care accounting for the greatest part. Greater severity of the disease (CDR), increased direct medical, social care, informal care, and total costs.
Gerves et al. (2014) France [56]	Cross-sectional, economic evaluation	Community-dwelling; 79; MMSE 19	OLS, two-stage least square regression	57 dyads	MMSE	Living with the PwD, severity of dementia and hours spent on formal care were significantly associated with informal care time.
Åkerborg et al. (2016) Sweden [57]	Cross-sectional, economic evaluation	Community-dwelling; min 79; MMSE 17	GLM, cost-estimation	296	MMSE	Cost of dementia care increased with dependence and the impact of other disease indicators was mainly mediated by dependence.
Boström et al. (2007) Sweden [50]	Cross-sectional, economic evaluation	Community-dwelling and nursing home; 78; DLB and AD diagnose	Stepwise linear regression	34 DLB, 34 AD	Formal DLB and AD diagnose	Dependency in instrumental activities of daily living was strongly correlated with resource use in DLB patients.
Carter et al. (2019) Ireland [55]	Economic evaluation	Community-dwelling; 82; > 50% severe dementia g)	t-tests, cost-estimation	42	Dementia Severity Rating Scale (DSRS)^g^	Keeping highly dependent home-dwelling PwD is not cheap and raises questions about optimal resource allocation on the boundary of home care and residential care.
Ersek et al. (2010) Hungary [58]	Cross-sectional, economic evaluation	Community-dwelling; 77.4; MMSE 16.7	Cost-estimation	88	MMSE	Dementia related costs were much lower in Hungary compared to Western European countries. From the societal point of view, there was no remarkable difference between the costs of PwD living at home and in nursing homes.
Farre et al. (2018) Spain [59]	Cross-sectional, economic evaluation	Community-dwelling and nursing home; 83.1 (65); MMSE 15.1	Cost-estimation	287	MMSE	Cognitive impairment contributed to the cost of lost labour productivity in informal caregiver, especially in home care.
Gustavsson et al. (2011a) Cross-country^a^ [62]	Cross-country/cross-sectional	Community-dwelling and nursing home; 80.8; median MMSE stage was mild	OLS, cost-estimation	1222	MMSE, AD patients	ADL-ability was the most important predictor of societal costs of care in community dwellings irrespective of country and should therefore be central in the economic evaluation of Alzheimer’s disease therapies.
Gustavsson et al. (2011b) Cross-country^b^ [60]	Cross-sectional, economic evaluation	Nursing home; 75 (65.5); MMSE 20.7	t-tests, correlation, cost-estimation	2744	MMSE	Informal care was the most important component of costs of care in a mild-to-moderate Alzheimer clinical trial sample, and it was primarily driven by the ADL-ability.
Gustavsson et al. (2010) Cross-country^c^ [61]	Cross-sectional, economic evaluation	Nursing home; 76.3; MMSE 20.4	GLM with log, cost-estimation	1381	CDR, MMSE	ADL was an important determinant of care costs. Formal care service use was lower and informal care higher in Southern Europe compared to Western and Northern Europe.
Vossius et al. 2019 Norway [49]	Cross-sectional, longitudinal, economic evaluation	Community-dwelling; 81.5; CDR-SoB 6.4^h^	GLM, cost-estimation	257	MMSE, CDR	There is no potential cost-saving effect of day care designed for people with dementia. The use of day care did neither result in a reduced use of care nor in a delay of nursing home admission.
Wubker et al. 2015 Cross-country^d^ [63]	Cross-country, economic evaluation	Community-dwelling; 83.3; SMMSE mild^i^	OLS, cost-estimation	1661	MMSE	Transition into nursing home, increased total costs of dementia care from a societal perspective.
Handels et al. 2018 Cross-country^e^ [64]	Cross-sectional, economic evaluation	Community-dwelling; 78 (66); MMSE 19	OLS, cost-estimation	451	MMSE	The study found varying relationships between unmet needs and quality of life, and no association between unmet needs and care costs, although the results were sensitive to various factors.
Costa et al. (2018) Cross-country^f^ [65]	Cross-sectional, economic evaluation	Community-dwellig, nursing home; 83.2 (63); MMSE moderate-severe	Cost-regression	1446	Formal diagnose, MMSE	Agitation symptoms had a substantial impact on informal care costs in the community care setting.
Buylova et al. (2020) UK [73]	Cross-sectional, economic evaluation	Nursing home; (62) FAST; 6e and above	OLS, cost-estimation	79	DSM-IV	Agitation was a key driver of costs in people with advanced dementia presenting complex challenges for symptom management, service planners, and providers.
Vandepitte et al. (2020a) Belgium [67]	Cross-sectional, economic evaluation	Community-dwelling; 78.7 (67.4); median GDS stage moderate-severe	OLS, cost-estimation	355	GDS	Characteristics of the caregiver and the PwD were associated with the monthly costs of care from a third-party payer and a societal perspective.
Vandepitte et al. (2020b) Belgium [54]	Cross-sectional, economic evaluation	Community-dwelling; 78.7 (67.4); median GDS stage moderate-severe	Cost-effectiveness analysis based on modelling	355	GDS	In-home respite care program in addition to standard community-based dementia care was a cost-effective approach compared with standard community-based dementia care.
Hojman et al. (2017) Chile [53]	Cross-sectional, economic evaluation	Community-dwelling; median age range 61–80 (60.7); Mean ADL; 62.5	GLM regression, cost estimation	330	SS-IQCODE	Lower socio-economic status was associated with higher costs due to informal care and, possibly, symptom severity.
Nakabe et al. (2019) Japan [51]	Cross-sectional, online survey	Community-dwelling; 81.8 (52.2); median care-need level: 2	χ^2^ automatic interaction detection (CHAID) analysis	1383	Own estimation of care-need levels based on function	Informal care costs were related to caregivers’ employment and cohabitation status rather to the situations of people with dementia. Out-of-pocket payments for long-term care services were related to care-need levels and family economic status.
Nakabe et al. (2018) Japan [52]	Cross-sectional, Online survey	Community-dwelling, nursing home; 82.5 (51.9); median care-need level: 2	Descriptive analyses	3841	Own estimation of care-need levels based on function	The inclusion of informal care costs reduced the differences in total personal costs among the residence types.

^a^ Sweden, Spain, UK, US, ^b^ Australia, France, HK, Italy, Netherlands, NZ, Singapore, US, ^c^ Sweden, Denmark, UK, Belgium, France, Germany, Switzerland, The Netherlands, Italy, Spain, Greece, Romania, ^d^ Estonia, Finland, France, Germany, the Netherlands, Spain, Sweden and the UK, ^e^ Germany, Ireland, Italy, the Netherlands, Norway, Portugal, Sweden, UK, ^f^ Estonia, Finland, France, Germany, Netherlands, Spain, Sweden and England, ^g^ Carter et al. [55], page 5, ^h^ SoB - sum of boxes. Vossius et al. [49], page 6, Table 1, ^i^ SMMSE standardized mini-mental state examination, Wübker et al. [63], page 696

**Table 4:** Economic evaluations using RUD

Author, year, country, source number	Type of health care system (insurance)	Valuation year, currency, unit	N	Cost variables identified	Perspective	Total societal cost	Informal cost/total societal cost (%)
Darba et al. (2016) Spain [68]	Public	2013, EUR, monthly	343	Formal care, medical costs, informal care, indirect costs	Individual societal	5362	
Michalowsky et al. (2018) Germany [72]	Public	2015, EUR, monthly	425	Formal cost, medical care, informal care, indirect	Individual and societal perspective	2156.4	71%
Åkerborg et al. (2016) Sweden [57]	Public	2012, EUR, monthly	170	Medical care, formal care, informal care	Individual	3604.10	9.3%
Boström et al. (2007) Sweden [50]	Public	2005, EUR, monthly	DLB: 34, AD: 34	Medical care, formal care, indirect costs	Societal	DLB: 28974.3, AD: 14079.6	DLB: 26.9%, AD: 39%
Carter et al. (2019) Ireland [55]	Private	2017, EUR, monthly	42	Medical, formal care, informal care	Individual		24.3%
Ersek et al. (2010) Hungary [58]	Public	2008, EUR, monthly	74	Medical, formal care, informal care	Individual, Societal	1649	17%
Farre et al. (2018) Spain [59]	Public	2017, EUR, monthly	287	Indirect cost (loss of labour working hours)	Societal	HC: 411.5, LTIC: 326.3	
Gustavsson et al. (2011b) Cross-country [60]		2006, GBP, annual	2744	Formal care, informal care	Societal	Mild: 9308 (10,924; 13,353) Mod.: 13980 (16,408; 20,055, Sev.: 19957 (23,422; 28,629)	87–93%
Gustavsson et al. (2011a) Cross-country [62]		2007, GBP, monthly	1222	Formal care, medical costs, informal care	Societal	HC: 15786, LTIC: 30037	30–60%
Gustavsson et al. (2010) Cross-country [61]		2006, EUR, monthly		Formal care, medical costs, informal care	Individual, societal	NE: 505, WE: 690, SE: 587.5	NE: 47%, WE: 36.9%, SE: 77.9%
Vossius et al. (2019) [49] Norway	Public	2017, EUR, monthly	257	Formal care, medical costs, informal care	Societal	8546	33.4%
Costa et al. (2017) Cross-country [65]		2014, EUR, monthly	1997	Formal care, medical costs, informal care, indirect costs	Societal	HC: 445, LTC: 561	HC: 73%, LTC: 53%
Gerves et al. (2013) France [56]	Public	2014, EUR, monthly	53	Formal care, medical costs, informal care, indirect costs	Individual, societal	4288.31	77.4%
Wubker et al. (2014) Cross-country [63]		2010, EUR, monthly		Formal care, medical costs, informal care, indirect costs	Societal	Estonia: 702.2, the Netherlands: 2450.6, Sweden: 2225	Estonia: 68.2%, the Netherlands: 30.8%, Sweden: 48.3%
Handels et al. (2018) Cross-country [64]		2015, EUR, monthly	451	Formal care, informal care, indirect costs	Societal	17,296 (16634–18,004)	66%
Vandepitte et al. (2020a) Belgium [67]	Public	2018, EUR, monthly	355	Formal care, informal care, indirect costs	Societal, third party payer	2338	45%
Buylova et al. (2020) UK [73]	Private and public	2012, GBP, monthly	79	Formal care, informal, indirect costs	Societal	40,606	29%
Nakabe et al. 2018 Japan [52]	Public	2016, USD, monhtly	330	Informal care, indirect costs	Individual		
Nakabe et al. 2019 Japan [51]	Public	2016, USD, monthly	1383	Informal care, indirect costs	Individual		
Hojman et al. 2017 Chile [53]	Private	2009, USD, annual	3841	Formal care, medical costs, informal care, indirect costs	Societal	17,599	75

Information on the share of informal care costs to total cost is authors’ own calculations. In Bostrom et al. [50] informal care added as lost production of workers and lost leisure of retired persons, and costs were given according to the following groups: Dementia Lewy Body (DLB) and Alzheimer’s Disease (AD). In Gustavsson et al. [61] costs are divided regionally: North Europe (NE), Western Europe (WE) and Southern Europe (SE). In 5 studies costs were given annually, in 2 studies costs were given weekly and further, 2 studies reported costs by 6 months. All costs were transformed into monthly cost. Currency abbreviations: Euro (EUR), US dollar (USD), Great British Pound (GBP)

#### Characterization of dementia

The cost studies included in Table 3 diverge regarding dementia etiology and assessment of cognitive and functional level. Thirteen studies [50, 51, 56–65] used the MMSE scale to define cognitive ability, one study, by Carter et al. [55] used the Dementia Severity Rating Scale (DSRS) [66], two studies, by Vandepitte et al. [54, 67] used the GDS scale, and one study, by Darba et al. [68] used the Clinical Dementia Rating Score (CDS) [69]. Five studies included patients with Alzheimer’s Disease (AD) diagnose [56, 60–62, 68], while Boström et al. [50] included patients with AD and Lewy Body Dementia (DLB). Nakabe et al. [51, 52] used their own estimation of care-need levels based on the ADL self-maintenance scale by Katz [70]. Hojman et al. [53] used the SS-IQCODE (Short Spanish version of the Informant Questionnaire of Cognitive Decline in the Elderly) [71].

#### Perspective of the cost–analyses and disaggregation of costs

Eighteen of the 21 included economic evaluations in Table 3 used a societal level perspective. Seven of the studies [54, 56, 58, 61, 67, 68, 72] combined both individual and societal perspectives and three [51, 52, 57] had individual level perspective. Two studies, i.e., Vandepitte et al. [54, 67] included cost perspectives from a third-party payer. Sixteen COI studies provided cost information for formal and informal care and direct medical care (out-patient, in-patient treatment, medication and/or emergency visit). All studies except for one, by Ersek et al. [58] provided indirect costs. Åkerborg et al. [57] and Nakabe et al. [51, 52] did not include costs of medication treatment for PwD nor did the two latter studies include formal costs. Costs for accommodation constituted a large share (68% of total care) in Åkerborg et al. [57]. Handels et al. [64] provided data on hospitalization of caregivers and was thus the only study providing health care costs of caregiver.

#### Valuation of unit cost

Following Wimo et al. (2013) [1], 17 studies in Table 3 used the opportunity cost method for both employed and retired caregivers. All studies used 35% of average income to value lost leisure time for retired caregiver. One study, by Ersek et al. [58] did not calculate indirect costs, and two studies, i.e. Wubker et al. [63] and Darba et al. [68] used the replacement cost methods. One study, by Hojman et al. [53] used both opportunity and replacement costs. All studies used hours per month, except Åkerborg et al. [57] who used days. Buylova et al. [73] was the only study providing weekly costs.

#### Estimation of resource consumption and sources of activity data

Twelve of 21 studies listed in Table 3 used all three informal care items (number of hours per month on instrumental and personal activities of daily living, and supervision) of the RUD questionnaire [52–54, 56, 58, 60–64, 67, 73], interviewing caregivers. Three studies in Table 3 used RUD Lite [56, 60, 61, 68, 73] Eight studies excluded supervision [49, 50, 52, 55, 68, 72]. All studies, except two [59, 68], reported cost details of informal care and thus, making estimations of the share of informal costs to total costs possible (Table 4, last column). Because most COI studies differed in their choice of valuation year, currency, and unit cost (Table 3) there is high uncertainty about the shares. In general, informal care varies with a range of 21–33% concerning different valuation methods [10, 19]. Because of this, we mostly emphasized the share of informal costs to total cost. The largest share in the Nordberg study (2005) was supervision, which may in some cases overlap with ADL and IADL. The data source was provided fully by all studies, but four studies [49, 57, 59, 73] did not provide substantial discussion and details of the three items of RUD despite using all components. Of these, only Vossius et al. [49] excluded supervision. Neubauer et al. [36] and Darba et al. [68] were the only studies that also included costs of the secondary caregiver. In a validation study by Neubauer [36], it was found that 93% of the caregivers yielded complete and plausible feedback on the number of hours of informal care [44]. Three studies [58, 63, 72] still emphasized possible errors of recall bias and the possibility of overlap between supervision on the one hand and IADL and ADL based care on the other. These studies used a maximum threshold of number of hours of supervision or IADL and ADL based caregiver hours.

#### Discounting costs and sensitivity analyses

Since most of the economic evaluations in Table 3 are cross-sectional, there is no discounting of costs. Vossius et al. [49], however, had a follow up of 12- and 24 months. The cost-effectiveness study of the in-home respite care treatment of both caregivers and PwD [54, 74] used the annual discount rate (beyond the first year of the model) of 3% for future costs and 1.5% for future QALYs recommended by the Belgian Healthcare Knowledge Centre. Only 10 of 21 economic evaluations listed in Table 3 performed sensitivity analyses [53–55, 58, 59, 63–65, 67, 72]. Hojman et al. [53] and Michalowsky et al. [72] used an alternative valuation method of informal care as sensitivity analysis.

#### Presentation of results and inclusion of all issues of concern

All 21 studies included in Table 3 provided a careful explanation of the methods adopted. However, one study fulfilled this only partly, i.e. Bostrom et al. [50]. Moreover, included studies also provided an analysis of issues of concern, while only partly fulfilled by the following studies [49, 58–60, 62]. The cost-effectiveness analysis of the RCT study by Vandepitte et al. [54] used the control group to provide analysis of competing alternatives. The control group however, had treatment as usual and not alternative care which makes the results more indirect [33, 75]. Twelve studies provided sources of previous studies, epidemiological approaches, or had a discussion of the problems regarding an absence of previous epidemiological studies. Five studies did this partly [49, 58–62]. Darba et al. [68] mentioned the limitation of not comparing PwD with older adults without dementia. A strength in the design of Boström et al. [50] is the comparison between AD and DLB, yet, the strength is limited by a small sample size of patients with DLB (*n* = 15). A strength of Carter et al. [55] is the comparison between public and private residential care. While Ersek et al. [58] provided national estimates, the large sample size of both Nakabe et al. [51, 52] studies is due to an online survey design.

## Discussion

Our primary aim was to identify factors related to PwD and their caregivers, that are strongly associated with high caregiver burden and increased societal costs. We also aimed to determine specific health service interventions that reduce caregiver burden and societal cost levels, and to assess the methodological quality of the economic evaluations of RUD studies. This systematic review illustrates that the functional dependency and severity of dementia are strongly associated with increased monthly hours of care and societal costs, lasting over time. It is further demonstrated that programmes aimed at alleviating caregivers’ burden (e.g., the in-home respite care “Baluchonnage”) may be more cost-effective and increase QALYs in an RCT study design. Since 2000, only two RCT studies on care burden assessed by RUD are conducted. However, low sample sizes and short follow-up period encourage researchers to conduct further explorations.

The results of this article are of key importance for society, politicians, and clinicians due to the expected increasing costs of dementia, and the pressure on family caregivers and the primary health care system, in general. While the need for care will continue to increase, the number of potential informal caregivers will stagnate [5]. The results are also crucial when designing new treatment and health care service innovation.

Michalowsky et al. [72] is the only economic evaluation study to fulfil all points of the Drummond criteria, and further, Darba et al. [68] fulfils 9/10. The two studies showed the importance of disease progression to caregiver burden and societal cost. Darba et al. [68] identified that the severity of dementia was not only associated with RUD outcomes in terms of hours per month, but also with all cost components including total costs for the society. The fact that functional dependency and severity of dementia are cost-intensive is also in accordance with previous research [76]. For instance, a study by Schwarzkopf et al. [77] investigated the cost differences between mild and moderate dementia and found that informal care costs of moderate dementia exceed the costs of mild dementia by 69.9%, whereas costs for formal health care services differ by 14.3% between the two dementia stages. Furthermore, caregiver factors such as the level of education are also associated with high societal cost due to the alternative costs and third-party payers’ cost (i.e., government agencies or employers). The total costs also varied significantly across socio-economic status of PwD, in which persons with lower status had higher costs [53]. Finally, single neuropsychiatric symptoms such as depression [40] as well as the total symptom burden [35] generated a high amount of caregiver hours per month.

The findings of Hojman et al. [53] demonstrate the importance of differences in costs and resource use of the dementia disease across groups of PwD. However, what the review reveals is that few economic evaluation and resource use studies analyze costs and resource use across groups of caregivers. Two studies with an online sample, and thus a young population of caregivers have done so the last 20 years [51, 52]. Moreover, among the evaluated studies, health status as well as comorbidities of caregivers and the influence on the total cost and resource use are absent, and in COI studies, medical care costs are mainly comprised by the medical care cost of PwD. Previous studies have shown a positive relation between caregiving intensity on the one hand and adverse health effects and increased health care utilization on the other [78]. As age is associated with higher medical care costs among PwD [76], different age groups among caregivers affect resource use and costs. Future economic evaluation studies and cost of illness studies should more thoroughly include health care expenses of caregivers.

The MAKS intervention elaborated in Luttenberger et al. [39] - a multicomponent non-pharmacological therapy comprising of motor stimulation, activities of daily living, and cognitive stimulation for nursing home residents (*n* = 71) which was delivered for 6 months, 6 days a week, 2 h a day [39] with an objective to measure informal care burden and dementia symptoms for PwD in nursing homes had no significant effect on informal care hours, compared to the control group. On the contrary, the in-home respite care intervention study by Vandepitte et al. [54], where the intervention group comprising of home-dwelling PwD and caregivers received in-home respite care support in personal and instrumental ADL every 6 month for 5 days, concluded that their care programme was cost-effective and generated increased QALYs compared with standard community-based dementia care. In the respite program, caregivers could be relieved from their caregiver’s task for at least 5 days while a trained employee took their place. For the patient, all daily habits/activities and resource use remained unchanged.

According to our methodological analyses based on the GRADE guidelines and the Drummond criteria, some characteristics of dementia are more strongly associated with higher societal costs than others. The indirect costs and the total societal costs are higher for home-dwelling PwD compared to nursing home dwellers, especially those living alone. Importantly, the *direct* costs were higher when living in a nursing home. These cost differences were also found in previous non-RUD based studies [21, 27]. High costs and resource use were associated with disease severity, functional dependency, and BPSD. It is unclear what type of dementia etiology demands more resources and generate higher costs, but persons who have had the diagnosis for a long period are especially resource intensive. Interventions aimed at home-dwelling PwD, in combination with standard care could relieve the burden for caregivers and increase safety for PwD.

The divergent use of the outcome measurement RUD may potentially under- or overestimate informal care hours, cost levels, and the cost-effectiveness. Although 17 of 26 of the included studies used all three measurements - ADL, IADL and supervision - inclusion of supervision may overlap with ADL and IADL due to recall bias [44]. Similarly, excluding supervision may underestimate care time and costs [29]. Only three studies used a maximum threshold of number of hours to solve potential recall bias. Although the RUD instrument has enhanced comparability of COI studies and other non-cost RUD studies, the review calls for a more harmonized approach.

The clinical and economic importance is to provide knowledge that can lower the hours of care per month along with the direct and societal costs it generates for the family and formal caregivers.

Future studies that aim to reduce the burden of carers and the related societal costs should include randomized controlled trials with longer follow-up and compare different treatment approaches to each other as well as standard care; they should also investigate the incremental cost-effectiveness ratio of the treatment among both PwD living in the municipality and those living in nursing homes. The intricacy of alleviating the entire burden of caregivers demands for health service innovation [79]. However, as organization of health care involves multiple stakeholders and layers of organizational levels – a multicomponent complex interventions could potentially mitigate caregiver burden and costs [79–81]. Multicomponent intervention is not a discrete package of separate components, but a process of changing what complex systems do, introducing new ways of how the system can work [82]. The treatment in multicomponent intervention should also target disease severity, functional dependency, and BPSD, and distinguish between different caregiver profiles including their socio-economic status. The clinical and economic importance of the systematic review is to provide knowledge that can lower the hours of care per month and the direct and societal costs it generates by family and formal caregivers.

### Limitation

The criteria we set in this systematic review (Table 1) may potentially exclude important information. First, we set the criterion of only reviewing studies with PwD aged 65 or older, mainly because the degree of resource utilization and cost levels may be very different between young (aged 55 or older) and older PwD [10].

Oure systematic review focused exclusively on the resource use (time) variables. Although this focus enhances comparability of the findings, there are multiple measurements of informal caregiver burden. For instance, we did not consider subjective well-being of caregivers, even though this is highly emphasized in the literature. The Relative Stress Scale (RSS), a self-rated 15- item scale that measures the caregiver’s burden of care [83], offers an opportunity to differentiate between different patterns of caregivers distress, while Costa et al. [20] also show how multiple forms of informal care questionnaire are used.

The RUD questionnaire only allows loss of labour productivity information to be gathered from the perspective of the employee. Thus, its analysis requires the use of the human capital approach that may overestimate indirect cost. Alternative approaches exist when valuing productivity loss such as the friction period approach.

## Conclusion

The divergent use of the RUD components within RUD studies shows that future economic evaluations and observational studies should be better harmonized. There are only two RCTs with RUD as primary or secondary outcome, and only one of these shows a significant treatment effect. This calls for future RCTs to be more methodologically sound with appropriate sample sizes and a representative follow-up period. Cost-enhancing and resource intensive factors such as disease severity, functional level, and BPSD should be specifically targeted when designing future treatment or intervention for innovate primary health care services. Furthermore, new treatments should diversify between caregiver groups, and between different living situation of the PwD person with dementia.

## Data Availability

Data sharing is not applicable to this article as no datasets were generated during the current study.

## Abbreviations

AD: Alzheimer’s Disease
ADL: Activities of Daily Living
COI: Cost of illness
DLB: Dementia Lewy Body
EUR: Euro
GBP: Great British Pound
GLM: General linearized model
IADL: Instrumental Activities of Daily Living
ILTC: Institutional Long-Term Care
NPI: Neuropsychiatric Inventory
OLS: Ordinary least square
PwD: People with dementia
QoL: Quality of Life
RUD: Resource Utilization Dementia
RE: Random effectRUD-FOCA – Resource Utilization Dementia Formal Care
RUD Lite: Resource Utilization Dementia with a focus on patient’s resource use
USD: US dollar
